# Effects of Varying Nitrogen Sources on Amino Acid Synthesis Costs in *Arabidopsis thaliana* under Different Light and Carbon-Source Conditions

**DOI:** 10.1371/journal.pone.0116536

**Published:** 2015-02-23

**Authors:** Anne Arnold, Max Sajitz-Hermstein, Zoran Nikoloski

**Affiliations:** 1 Computational Systems Biology, School of Engineering and Science, Jacobs University Bremen, Campus Ring 1, 28759 Bremen, Germany; 2 Systems Biology and Mathematical Modeling Group, Max Planck Institute of Molecular Plant Physiology, 14476 Potsdam, Germany; RIKEN Center for Sustainable Resource Science, JAPAN

## Abstract

Plants as sessile organisms cannot escape their environment and have to adapt to any changes in the availability of sunlight and nutrients. The quantification of synthesis costs of metabolites, in terms of consumed energy, is a prerequisite to understand trade-offs arising from energetic limitations. Here, we examine the energy consumption of amino acid synthesis in *Arabidopsis thaliana*. To quantify these costs in terms of the energy equivalent ATP, we introduce an improved cost measure based on flux balance analysis and apply it to three state-of-the-art metabolic reconstructions to ensure robust results. We present the first systematic *in silico* analysis of the effect of nitrogen supply (nitrate/ammonium) on individual amino acid synthesis costs as well as of the effect of photoautotrophic and heterotrophic growth conditions, integrating day/night-specific regulation. Our results identify nitrogen supply as a key determinant of amino acid costs, in agreement with experimental evidence. In addition, the association of the determined costs with experimentally observed growth patterns suggests that metabolite synthesis costs are involved in shaping regulation of plant growth. Finally, we find that simultaneous uptake of both nitrogen sources can lead to efficient utilization of energy source, which may be the result of evolutionary optimization.

## Introduction

Metabolism can be regarded as an integrated network of biochemical reactions through which simple chemical building blocks are assembled into larger molecules supporting cellular tasks which ensure viability and reproduction. As a result, metabolism does not operate in isolation from the other levels of cellular organization, and its state is tightly regulated via the participating proteins that enable most of the underlying reactions [1]. In addition, metabolism responds timely to changes in the environment or internal cues orchestrated via regulatory cascades [2]. The metabolic state is then ultimately modified by reallocating the utilization of available (internal and external) resources, namely, the acquired simple precursors, the enzymatic composition, and the levels of intermediate building blocks.

Metabolic resource allocation strategies usually lead to beneficial adaptations of the metabolic state, which can be explained by a cost-benefit principle: resources are diverted towards other metabolic processes as long as they result in larger benefit, *e.g*., growth rate. Therefore, systematic evaluation of resource allocation strategies has the potential of elucidating principles underlying metabolic regulation. However, the cost-benefit analysis requires condition-specific quantification of metabolic costs.

Metabolite synthesis costs can be quantified by the (*de novo*) synthesis requirement for resources, which then cannot be allocated to other metabolic processes. Various definitions of metabolic costs have been proposed, reflecting the variety of possible resources required for synthesis, *e.g*., carbon [3], nitrogen [3] or energy [4–7]. Among all resources, energy is arguably the most important one, because it is not only needed for metabolite synthesis but also for driving the entirety of metabolic processes.

Amino acids, as building blocks of peptides and proteins, represent a major part of the cellular composition. Therefore, their synthesis costs in terms of energy are expected to play an important role in energy allocation. By constituting enzymes, amino acids are directly involved in enabling metabolic functionality, including resource (re)allocation. Moreover, amino acids can be seen to bridge carbon and nitrogen metabolism in plants, of which they are integral components [8].

Various studies examined amino acid synthesis costs by using different methodologies [4–7, 9, 10], predominantly aiming at energetic costs in terms of ATP. Based on the quantification of amino acid synthesis costs, cost-minimization was suggested as a principle shaping protein expression, which could explain the observed utilization of amino acids with low energetic costs in highly expressed genes for various bacteria [5, 6] as well as for other organisms [9].

Plants use two energy sources—sunlight and storage molecules—to drive their metabolism, and the utilization of either is expected to affect amino acid synthesis costs. Sunlight is used in photosynthesis to generate energy equivalents and to fix carbon during day, including storage molecules (*e.g*., starch). Plant photosynthetic cells during night, and plant non-photosynthetic cells in general, have to utilize energy from storage molecules produced during day. The availability of energy source and its usage determines the energy budget of individual cells and the whole plant. Understanding the (re)allocation of the cellular energy budget under varying conditions would shed light on the cost-related regulation of plant metabolism.

Most plants can grow on different nitrogen sources, utilizing different assimilation pathways, which ultimately can be expected to affect amino acid synthesis costs. To this end, it was experimentally shown for *Arabidopsis thaliana* (*Arabidopsis*) that the nitrogen source, predominantly nitrate (${\text{NO}}_{3}^{-}$) and/or ammonium (${\text{NH}}_{4}^{+}$), influences amino acid and protein levels [11] as well as growth rate [11, 12]. While the cost-minimization hypothesis may be used to explain these observations, model- or data-driven testing of the hypothesis is required.

Here, we present a model-based methodology to determine condition-specific amino acid synthesis costs based on flux balance analysis, which builds on and improves an existing cost measure [7]. The novel cost measure accounts for the model-dependent capability to produce energy equivalents from different energy sources, as well as for differences in energy source consumption and utilization during amino acid synthesis. By using the proposed cost measure, we determine amino acid synthesis costs in *Arabidopsis* in terms of ATP and examine how they may be affected by changes in environmental conditions and cellular scenarios.

Since the results may depend on the characteristics of the employed metabolic reconstruction, we conduct a comparative analysis of amino acid synthesis costs in *Arabidopsis* by applying the proposed measure to three models—of Poolman *et al*. [13], de Oliveira Dal’Molin *et al*. [14], as well as Arnold and Nikoloski [15]. This enables the examination of costs with minimial bias due to errors and shortcomings of individual models.

As our major contribution, we rigorously examine the effect of differences in nitrogen supply as well as of autotrophic versus heterotrophic growth conditions on individual amino acid synthesis costs. We elucidate the effect of nitrogen supply by examining four different scenarios: sole ${\text{NO}}_{3}^{-}$ supply, sole ${\text{NH}}_{4}^{+}$ supply, as well as supply of both nitrogen sources with an additional constraint for equal uptake (“equal nitrogen source uptake”) and without (“arbitrary nitrogen source uptake”). The latter results in a self-adjusting uptake ratio towards optimal energy source utilization. The analysis is carried out for day and night scenarios by integration of known regulatory effects on specific enzymes.

## Results and Discussion

Following a description of our method to determine amino acid synthesis costs, we first examine the differences of amino acid costs due to the employed metabolic network model. Second, we explore the effect of day and night as well as cellular growth conditions on amino acid synthesis costs. In particular, we examine the two major cell types with respect to their trophic level: carbon-fixing cells using light energy, photoautotrophs, and cells utilizing organic compounds, namely, glucose (Glc), as both carbon and energy source, namely chemoheterotrophs. In the following, we refer to these as autotrophic and heterotrophic cells, respectively. Obviously, the night scenario is also heterotrophic. Third, we analyze the influence of available inorganic nitrogen sources on amino acid costs. In accordance with earlier studies [4, 5, 9, 16], the costs of the different amino acids fall in a wide range from 11 to 129.2 ATP per amino acid. Nevertheless, the costs largely differ across the used models, examined environmental and cellular scenarios, as well as the different nitrogen sources. All calculated costs are to be found in the Supporting S1 dataset.

### Calculation of amino acid synthesis costs

We quantify synthesis costs of amino acids in terms of the metabolic energy equivalent ATP. The energetic equivalency of ATP can be identified with the Gibbs free energy which is consumed during ATP formation from ADP and inorganic phosphate (Pi) or released via ATP hydrolysis. For simplicity, we refer to these processes as “production of ATP” and “consumption of ATP”, respectively. As ATP hydrolysis can drive various endothermic reactions by providing free energy and because other energy equivalents (*e.g*., the redox equivalent NAD(P)H) can be utilized for ATP production, we refer to ATP as the universal cellular energy currency.

We define the cost of an amino acid as the energy required for its *de novo* synthesis in terms of ATP. In accordance with Craig and Weber [4], cost is quantified as the amount of ATP sacrificed by diverting energy source to amino acid synthesis instead of ATP production. The definition differs from that used by Kaleta *et al*. [16], which is based on ATP consumption starting from energy source, implying that allocation of the resource to amino acid synthesis does not impose a burden on the remaining metabolism. We distinguish between different carbon, nitrogen, and energy sources pertaining to the environmental and cellular scenarios of interest. To this end, we utilize available metabolic reconstructions together with flux balance analysis [17] to examine energy-efficient amino acid synthesis pathways under specific conditions and determining synthesis costs. We denote such metabolic reconstructions as “structural metabolic models”, since they usually encompass only structural information.

Flux balance analysis considers the system to be at a (pseudo) steady-state, and neglects reaction kinetics. While this drastically simplifies the description of metabolism, the reduced requirements (*i.e*., reaction stoichiometries, reaction directions and upper/lower flux boundaries) enable the examination of genome-scale metabolic networks, successfully applied in numerous studies [18, 19] (see Materials and Methods). In contrast, reliable kinetic modeling is usually limited to small network sizes due to lack of knowledge about reaction kinetics, metabolite concentrations and kinetic parameters, preventing its application to reliably determine amino acid synthesis costs.

The (pseudo) steady-state is a reasonable assumption in our case, since we are not examining short-term dynamics of amino acid synthesis, but rather deal with static conditions approximately valid for several hours. Environmental conditions are integrated by constraining influx of nutrients or other resources, *e.g*., photons. The reduction upon steady-state fluxes does exclude the incorporation of dynamic effects, for instance, resulting from different day lengths. Moreover, the flux balance approach cannot account for complex metabolic behavior, such as reaction to high (toxic) levels of metabolites/ions.

Our algorithm to determine individual amino acid synthesis cost is based on optimization of a structural metabolic model (using flux balance analysis) and proceeds in three steps (see figure in section Material and Methods), determining: (i) maximum of ATP production per energy source uptake (ATP production efficiency), (ii) minimum energy source uptake per unit amino acid synthesis (energy source requirement), and (iii) maximum surplus ATP production while synthesizing the amino acid (surplus ATP cost). The outcomes are integrated to determine individual amino acid synthesis costs. The total ATP cost of amino acid synthesis is calculated from step (i) and (ii), as the product of the energy requirement and the ATP production efficiency. It quantifies how much ATP could be produced from the consumed amount of energy source instead of using it for amino acid synthesis, integrating the model-specific capability to produce ATP from an energy source.

The total ATP costs can be larger than the amount of energy eventually utilized in amino acid synthesis for two reasons. First, the energy source may also serve as the carbon source, *e.g*., Glc in heterotrophic cells. In this case, it can occur that more Glc is taken up than needed for providing energy to drive amino acid synthesis, because the demand for Glc to build the carbon skeletons exceeds the demand for Glc for energy allocation. Second, inflexibilities of the system could render it incapable to optimally utilize the energy source, *e.g*., due to fixed stoichiometries in the generation of ATP and NAD(P)H (a detailed discussion of the issue is given in a subsequent section). In both cases, while synthesizing the amino acid with minimum energy source uptake, a surplus of ATP can be generated and diverted to other processes. The energetic costs of amino acid synthesis are then calculated as the difference of the total and the surplus ATP costs. A detailed description of the three steps of our algorithm is provided in the Material and Methods section.

### Model comparison

The metabolic models of Poolman *et al*. [13], de Oliveira Dal’Molin *et al*. [14], and Arnold and Nikoloski [15] represent the state-of-the-art structural metabolic models of *Arabidopsis*’ primary metabolism. The model of Poolman *et al*. describes heterotrophic leaf cells, whereas the models of de Oliveira Dal’Molin *et al*., and Arnold and Nikoloski are capable to simulate both heterotrophic and autotrophic growth. The models of Poolman *et al*. and de Oliveira Dal’Molin *et al*. are both top-down reconstructions, starting from genome annotations. In contrast, the model of Arnold and Nikoloski is a bottom-up reconstruction, whereby the operability of the incorporated reactions and metabolites is ensured by starting from well-documented and necessary biochemical pathways. Therefore, the models differ in the total number of reactions and metabolites. Nevertheless, the size of the underlying operational network, which excludes blocked reactions and dead-end metabolites, is comparable (Table 1). Unique reactions and metabolites correspond to the numbers of different reactions and metabolites across all compartments. As the model of Poolman *et al*. comprises equivalence reactions, *e.g*., interconverting two species of fumarate (‘FumEquiv’), we do not provide the numbers of unique reactions and metabolites in this case.

**Table 1:** Properties of utilized networks.

Property	**Poolman**		**de Oliveira**		**Arnold**	
Reactions (unique)	1406		1601	(1472)	549	(345)
– Blocked reactions	0712		0956		0	
– Functional reactions (unique)	0694		0645	0(578)	549	(345)
— Importer and exporter	0039	(39)	0018	00(18)	097	0(37)
Metabolites (unique)	1253		1736	(1508)	407	(236)
– Dead-end metabolites	0428		0797		0	
– Functional metabolites (unique)	0825		0939	0(752)	407	(236)
Compartments (pseudo)	0001	0(1)	0004	000(3)	004	00(2)
– Transporter (unique)	–		0080	00(42)	125	0(87)
– Blocked transporter	–		0015		0	
– Functional transporter (unique)	–		0065	00(36)	125	0(87)

Comparison of the models of Poolman *et al*. [13], de Oliveira Dal’Molin *et al*. [14], and Arnold and Nikoloski [15] with respect to the network properties pertaining to operability.

The model of Poolman *et al*. is uncompartmentalized, although it contains a pseudo-compartment including a small number of segregated metabolites without any corresponding reactions. These metabolites are labeled by ‘mit’ and comprise NAD, NADH and protons which are participating in the TCA cycle and oxidative phosphorylation. In contrast, the models of de Oliveira Dal’Molin *et al*., and Arnold and Nikoloski contain the four major cell compartments: cytosol, chloroplast, mitochondrion and peroxisome. In addition, the model of de Oliveira Dal’Molin *et al*. includes three pseudo-compartments, namely the vacuole, an accumulation compartment, and a biomass compartment, to enable specific export. The model of Arnold and Nikoloski contains two subcompartments: the lumen in the chloroplast and the intermembrane space in the mitochondrion, which enable the generation of proton motive force needed for ATP formation. Pseudo-compartmentalization may cause the occurrence of futile cycles, which is observed in the case of the model of Poolman *et al*., prohibiting the analysis of ATP production in the original model definition. To resolve this issue and to further allow a meaningful comparison across the different models, individual modifications were applied, which are described in the Material and Methods section.

The interpretation of metabolite synthesis costs in terms of energy are expected to depend on the overall energetic expenditure of an organism into individual metabolite synthesis processes. Therefore, instead of comparing only the synthesis costs, we examine the ratios of total energetic investment into amino acids of *Arabidopsis* bound in proteins to obtain biologically meaningful results. To this end, we determine individual amino acid frequencies in *Arabidopsis* leaf proteins based on the dataset of Mooney *et al*. [20] (shown in Supporting S1 dataset), and associate them with the respective amino acid synthesis costs. It has to be noted that we cannot ascertain the available nitrogen source(s) in the underlying data set and that it is unclear if the examined cells were strictly autotrophic or heterotrophic (most likely both cell types were present). Therefore, we analyze amino acid costs not only for each model but also for the different trophic levels and nitrogen uptake scenarios. We consider amino acid synthesis costs at day which is in accordance with the experimental conditions of the utilized data set.

We calculate first the average weighted amino acid costs in a specific scenario according to

$$\langle C\rangle =\sum_{i=1}^{n}f\text{​}\left(a_{i}\right)\cdot C\text{​}\left(a_{i}\right),$$ (1)

with *a*_*i*_ denoting the individual amino acids, *f*(*a*_*i*_) the corresponding amino acid frequencies, *C*(*a*_*i*_) the costs of the specific amino acid, and *n* the total number of amino acids. The model of Poolman *et al*. yields lowest average costs for each examined trophic level and nitrogen uptake scenario compared to the costs based on the other models (Table 2). This is most likely due to the absence of compartments in this model, leading to simplified synthesis pathways, such that some metabolite interconversions requiring additional ATP do not occur. For instance, according to AraCyc [21], GMP is synthesized in the cytosol starting from IMP which, in contrast, is synthesized in the chloroplast. The IMP itself cannot be transported across the membranes separating these two compartments. Therefore, IMP has first to be converted to AMP under consumption of energy, which is then converted back to IMP after crossing the membrane. Obviously, such conversions do not take place in the model of Poolman *et al*.. The models of de Oliveira Dal’Molin *et al*., and Arnold and Nikoloski are both compartmentalized and exhibit higher costs for each scenario compared to the costs based on the model of Poolman *et al*..

**Table 2:** Comparison of amino acid costs.

〈*C*〉	**Poolman**				**de Oliveira**				**Arnold**			
	${\text{NH}}_{4}^{+}$	**50:50**	${\text{NO}}_{3}^{-}$	**arb**	${\text{NH}}_{4}^{+}$	**50:50**	${\text{NO}}_{3}^{-}$	**arb**	${\text{NH}}_{4}^{+}$	**50:50**	${\text{NO}}_{3}^{-}$	arb
Het	28.19	28.19	28.19	28.19	31.41	42.85	54.89	31.41	31.13	38.83	46.8	34.44
Aut	–	–	–	–	43.76	54.92	66.09	43.76	42.06	50.6	60.51	46.75
〈*δ*〉	Poolman: Arnold				de Oliveira: Poolman				Arnold: de Oliveira			
	${\text{NH}}_{4}^{+}$	50:50	${\text{NO}}_{3}^{-}$	arb	${\text{NH}}_{4}^{+}$	50:50	${\text{NO}}_{3}^{-}$	arb	${\text{NH}}_{4}^{+}$	50:50	${\text{NO}}_{3}^{-}$	arb
Het	5.61	10.82	18.61	7.92	5.01	14.67	26.7	5.01	2.44	5	8.09	4.87
Aut	–	–	–	–	–	–	–	–	2.54	4.57	5.58	4.39
*τ*	Poolman: Arnold				de Oliveira: Poolman				Arnold: de Oliveira			
	${\text{NH}}_{4}^{+}$	50:50	${\text{NO}}_{3}^{-}$	arb	${\text{NH}}_{4}^{+}$	50:50	${\text{NO}}_{3}^{-}$	arb	${\text{NH}}_{4}^{+}$	50:50	${\text{NO}}_{3}^{-}$	arb
Het	0.67	0.68	0.72	0.6	0.75	0.73	0.69	0.75	0.88	0.87	0.96	0.77
Aut	–	–	–	–	–	–	–	–	0.93	0.89	0.94	0.89

Comparison of the models of Poolman *et al*. [13], de Oliveira Dal’Molin *et al*. [14], and Arnold and Nikoloski [15] with respect to weighted average amino acid cost, 〈*C*〉, and average weighted distance of amino acid costs, 〈*δ*〉, as well as Kendall rank correlation *τ* of the product of frequencies and costs (p-value < 0.05 in all cases). The trophic level scenarios, heterotrophic and autotrophic, are denoted by Het and Aut, respectively. The examined nitrogen source scenarios are sole ${\text{NH}}_{4}^{+}$ supply, equal uptake of ${\text{NH}}_{4}^{+}$ and ${\text{NO}}_{3}^{-}$ (50:50), sole ${\text{NO}}_{3}^{-}$ supply, and arbitrary nitrogen uptake (arb).

Next, we calculate the average weighted distance of amino acid costs,

$$\begin{array}{c} \langle \delta \rangle =\sum_{i=1}^{n}f\;\left(a_{i}\right)\left|C_{X}\;\left(a_{i}\right)-C_{Y}\;\left(a_{i}\right)\right|, \end{array}$$ (2)

which quantifies the similarity of the costs *C* obtained by utilizing two different metabolic networks, here denoted by *X* and *Y*, in a specific scenario (then *C*_*X*_(*a*_*i*_) denotes the synthesis cost of amino acid *a*_*i*_, determined by using the metabolic network *X*). The costs based on the models of de Oliveira Dal’Molin *et al*., and Arnold and Nikoloski are more similar to each other than to the costs based on the model of Poolman *et al*.. This is corroborated by the Kendall rank correlation coefficient with respect to the product of individual amino acid synthesis costs and frequencies (Table 2). These qualitative findings hold irrespectively of the trophic level and the nitrogen source uptake scenario, and may indicate that the costs obtained by the compartmentalized models of de Oliveira Dal’Molin *et al*., and Arnold and Nikoloski are more reliable.

### Comparison of day and night scenarios under autotrophic and heterotrophic growth conditions

Probably the best investigated environmental factor in plant research is the alternation of day and night which causes extensive changes in the operation of plant metabolism, including most likely also adjustments in amino acid biosynthesis [22–26]. The carbon acquisition at day, either from organic or inorganic carbon sources, is also expected to affect energy resource utilization and, hence, amino acid costs. Therefore, we examine amino acid synthesis costs for day and night conditions and the impact of autotrophic and heterotrophic scenarios. Since the model of Poolman *et al*. [13] does not comprise light reactions, the comparison of heterotrophic and autotrophic day conditions can be conducted only for the models of de Oliveira Dal’Molin *et al*. [14], and Arnold and Nikoloski [15]. We perform the analyses for all relevant inorganic nitrogen supply settings to arrive at conclusions which hold irrespectively of the nitrogen source.

In general, the costs of amino acids under heterotrophic day and night conditions are more similar to each other compared to autotrophic day conditions. While the deviation of absolute amino acid costs is distinctly smaller between heterotrophic day and night conditions (see Supporting S1 dataset), the rank correlations are very large for all models and scenarios. The correlations are stronger between heterotrophic day and night conditions (Table 3; the only exception is the equal uptake of nitrogen sources in the model of Arnold and Nikoloski). The corresponding costs are similarly downscaled compared to autotrophic day conditions. This is caused by utilization of Glc as energy and carbon source in the heterotrophic day and night scenarios, which indicates that amino acids are synthesized via similar pathways, in contrast to the autotrophic day scenario utilizing carbon dioxide (CO_2_) as carbon and photons as energy source.

**Table 3:** Association of amino acid costs.

*τ*	**Poolman**				**de Oliveira**				**Arnold**			
	${\text{NH}}_{4}^{+}$	**50:50**	${\text{NO}}_{3}^{-}$	**arb**	${\text{NH}}_{4}^{+}$	**50:50**	${\text{NO}}_{3}^{-}$	**arb**	${\text{NH}}_{4}^{+}$	**50:50**	${\text{NO}}_{3}^{-}$	**arb**
Het: Nig	0.94	0.94	0.94	0.94	0.96	1	1	0.96	0.97	0.91	0.98	0.95
Aut: Het	–	–	–	–	0.94	0.96	0.92	0.94	0.87	0.92	0.96	0.89
Nig: Aut	–	–	–	–	0.96	0.96	0.92	0.96	0.88	0.93	0.96	0.86

Association of amino acid costs pertaining to the individual models with respect to day-night shift and trophic level determined by Kendall rank correlation *τ* (p-value < 0.05 in all cases). In particular, the scenarios are heterotrophic and autotrophic day (Het and Aut), and night (Nig). The examined nitrogen source scenarios are sole ${\text{NH}}_{4}^{+}$ supply, equal uptake of ${\text{NH}}_{4}^{+}$ and ${\text{NO}}_{3}^{-}$ (50:50), sole ${\text{NO}}_{3}^{-}$ supply, and arbitrary nitrogen uptake (arb).

**Fig 1:**
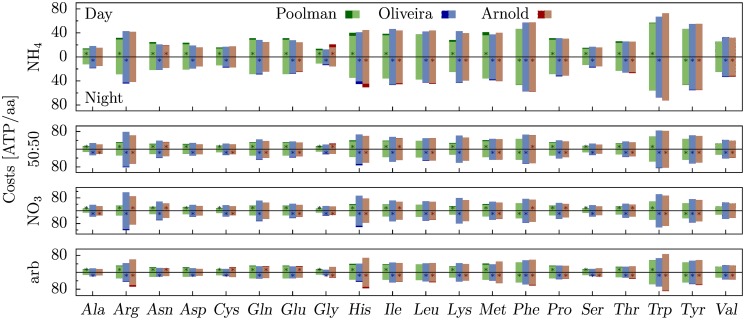
Amino acid costs for heterotrophic day and night conditions. Costs are based on the models of Poolman *et al*. [13] (green), de Oliveira Dal’Molin *et al*. [14] (blue), and Arnold and Nikoloski [15] (red). The light-colored bars represent the shared cost fraction of day and night, whereas the dark bars denote the cost difference. The scenario with the higher costs is additionally denoted by a star.

Analysis of costs based on the models of de Oliveira Dal’Molin *et al*., and of Arnold and Nikoloski, shows that amino acid synthesis costs pertaining to night conditions are in most cases larger than those for heterotrophic day conditions (Fig. 1). We observe an increase of Glc import accompanied by increased ATP surplus production at night which indicates that the night-specific restrictions on metabolism result in a more inflexible system compared to the day condition. In contrast, the costs based on the model of Poolman *et al*. are smaller at night than during the day. The reason is a decreased capability of the model to produce ATP from Glc at night (*i.e*., a decreased ATP production efficiency with respect to Glc), namely, 28 ATP/Glc compared to 31 ATP/Glc at day. Hence, utilizing a specific amount of Glc for amino acid synthesis leads to a smaller sacrifice of potential ATP production at night compared to utilizing the same amount at day. This effect likely outweighs the increase in costs due to additional inflexibilities at night. The change in the ATP production efficiency with respect to Glc cannot be justified by biologically meaningful interpretation and renders the costs based on the model of Poolman *et al*. questionable. Such discrepancy of ATP production efficiency is not detected with the other two models.

**Fig 2:**
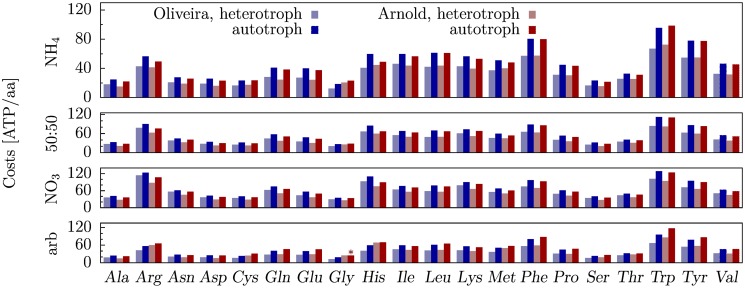
Amino acid costs for heterotrophic day and night (light) as well as autotrophic day (dark) conditions. Costs are based on the models of de Oliveira Dal’Molin *et al*. [14] (blue), and Arnold and Nikoloski [15] (red). The star denotes the exceptions for which autotrophic costs are higher than the corresponding heterotrophic costs.

The amino acid synthesis costs are higher under autotrophic compared to heterotrophic conditions (with exception of glycine synthesis in the model of Arnold and Nikoloski for arbitrary nitrogen uptake; Fig. 2). While synthesizing the common precursors for amino acid synthesis, *e.g*., 3-phosphoglycerate or pyruvate, starting from CO_2_ requires more energy than building these precursors from Glc, the resulting higher costs in autotrophic conditions are associated to the operating definition of synthesis costs. Here the cost of Glc utilization is given by the amount of ATP that would have been gained by its breakdown to CO_2_, which is necessarily smaller than the amount of ATP used in Glc synthesis (due to dissipative processes during anabolic synthesis).

The average weighted amino acid costs, incorporating individual amino acid abundances, based on the models of de Oliveira Dal’Molin *et al*. [14], and Arnold and Nikoloski [15] differ by more than ten ATP per amino acid on average for autotrophic versus heterotrophic day conditions (Table 2). This renders the incorporation of a plant’s trophic level with respect to specific cell types and external conditions an important factor in the analysis of energy-related metabolic trade-offs. Moreover, together with the observation that amino acids appear to be predominantly synthesized during day [27], this difference in costs leads to the following hypothesis: plant metabolism may have evolved towards more efficient amino acid synthesis during day, when energy from sunlight is available, which is reflected in lower amino acid synthesis costs on average for this condition.

It is important to note, that the implications of amino acid synthesis costs on energy resource allocation also depend on the energy budget. For autotrophic and heterotrophic cells as well as day and night conditions, mechanisms limiting the cellular ATP budget are inherently different, most likely leading to diverging budgets. Generally, drawing conclusions about resource allocation strategies solely from the comparison of amino acid synthesis costs for scenarios which exhibit different energy sources or supplies can be difficult.

### Effects of different nitrogen sources

The importance of an adequate nitrogen supply in the form of ${\text{NO}}_{3}^{-}$ and/or ${\text{NH}}_{4}^{+}$ is well-documented for optimal plant growth [11, 28]. For instance, Masakapalli *et al*. showed experimentally that amino acid and protein levels vary with the nitrogen source, although the measured net depletion of nitrogen from the medium was found to be of almost the same rate [11]. To examine whether the effects of nitrogen source on the absolute amino acid levels could be due to supply-dependent differences in synthesis costs, we investigate four nitrogen scenarios characterized by constraints on the nitrogen uptake: (i) sole ${\text{NO}}_{3}^{-}$ supply, (ii) sole ${\text{NH}}_{4}^{+}$ supply, (iii) equal uptake of the two nitrogen sources, ${\text{NO}}_{3}^{-}$ and ${\text{NH}}_{4}^{+}$, and (iv) arbitrary uptake of the two nitrogen sources, which results in a self-adjusting uptake ratio towards optimal energy source utilization.

The comparison of amino acid synthesis costs shows that scenarios in which nitrogen is assimilated exclusively in the form of ${\text{NO}}_{3}^{-}$/${\text{NH}}_{4}^{+}$ exhibit the highest/lowest individual costs (see Supporting S1 dataset). It is important to point out that the costs for the model of Poolman *et al*. [13] do not change across the nitrogen uptake scenarios (Tables 2 and 3), which is biologically unplausible (see below). Consequently, the following comparison is based on the models of de Oliveira Dal’Molin *et al*. [14], and Arnold and Nikoloski [15]. The difference of costs between sole supply of ${\text{NO}}_{3}^{-}$/${\text{NH}}_{4}^{+}$ is primarily due to additional energetic demands in the case of ${\text{NO}}_{3}^{-}$ assimilation, which requires conversion of ${\text{NO}}_{3}^{-}$ to ${\text{NH}}_{4}^{+}$ accompanied by oxidation of NADPH.

The average weighted amino acid synthesis costs show a difference up to 22 ATP per amino acid depending on nitrogen supply (Table 2). Such a difference is expected to play a significant role in the energy balance of plant metabolism and would offer an explanation for growth improvements when ammonium is introduced as nitrogen source additional to nitrate like it is observed for many plant species [29]. Plants often exhibit largest growth rate when a mixture of ammonium and nitrate is provided instead of sole ammonium supply [29], despite the energetic advantages of ammonium assimilation, which most likely is due to the toxicity of high concentrations for many plants [30]. While toxicity is an important aspect if ammonium concentrations are large, it cannot be incorporated in our cost measure, due to the modeling framework used, and has to be considered separately.

We find further corroboration that amino acid synthesis costs are associated with energy-related regulation in plants by inspecting the results of Masakapalli *et al*. [11]. The study shows decreased total assimilation of nitrogen into amino acids and proteins and a much higher carbon:nitrogen ratio for sole ${\text{NO}}_{3}^{-}$ supply compared to mixed nitrogen supply, which gives rise to the hypothesis that higher costs of amino acid synthesis result in lower synthesis rates. As mentioned above, the net depletion of nitrogen was observed at almost the same rate for the two scenarios, indicating that the nitrogen taken up by the plant is stored in another form, most likely as free nitrate in the vacuole [28]. Accordingly, the assimilated carbon (or Glc) which is not allocated to amino acid synthesis can be invested into other growth-related synthesis processes including, amongst others, the synthesis of sugars and organic acids. This is in accordance with the observed increased fresh weight growth rate and elevated levels of sugars and organic acids under sole ${\text{NO}}_{3}^{-}$ supply [11, 12].

The analysis of amino acid synthesis costs for arbitrary nitrogen source uptake reveals connections between nitrogen assimilation and energy source utilization. For the model of de Oliveira Dal’Molin *et al*., the costs and the nitrogen uptake match, as expected, with the minimum cost scenario (sole ${\text{NH}}_{4}^{+}$ supply). This is not the case for all amino acids in the model of Arnold and Nikoloski (see Supporting S1 dataset). Here, some amino acid synthesis costs exceed those in the minimum cost scenario and exhibit simultaneous utilization of ${\text{NO}}_{3}^{-}$ as well as ${\text{NH}}_{4}^{+}$, although the conditions would allow sole ${\text{NH}}_{4}^{+}$ uptake and, therefore, lower costs. This is due to the minimization of the respective energy source uptake in the cost calculation procedure (Material and Methods section): energy source uptake is smaller compared to sole ${\text{NH}}_{4}^{+}$ supply, but ${\text{NO}}_{3}^{-}$ utilization also results in a smaller surplus ATP production, eventually leading to higher costs. In the autotrophic scenario, the light reactions enable the utilization of light energy to generate NADPH and ATP. Thereby, in the case of sole ${\text{NH}}_{4}^{+}$ supply both energy equivalents are produced in a fixed ratio, which is 7:9 (NADPH:ATP) in the model of Arnold and Nikoloski. The final transfer of electrons to NADP is catalyzed by ferredoxin-NADP reductase (FNR; EC 1.18.1.2) which oxidizes ferredoxin. The well-established (but still highly discussed) hypothesis of the competition of FNR and nitrite reductase (NiR; EC 1.7.7.1) for reduced ferredoxin [31] offers an explanation for mixed nitrogen uptake under arbitrary uptake conditions. Accordingly, the assimilation of ${\text{NO}}_{3}^{-}$ will relax the fixed NADPH:ATP ratio by diverting reduced ferredoxin produced by photosystem I (EC 1.97.1.12) to nitrite reduction via NiR. Following the line of argumentation, assimilation of ${\text{NO}}_{3}^{-}$, ultimately, allows a more efficient usage of the energy source but at higher costs due to the higher energetic requirement for ${\text{NO}}_{3}^{-}$ compared to ${\text{NH}}_{4}^{+}$ assimilation. Similar argumentation holds for heterotrophic day and night conditions with respect to the fixed NADH:ATP ratio during Glc breakdown. Here, the FNR operates in the reverse direction such that NADPH reduces ferredoxin which is diverted to nitrite reduction. In accordance with the competition hypothesis, this would also result in a relaxation of the NADH:ATP ratio, since NADH can be easily converted to NADPH resulting in more efficient utilization of the energy source Glc.

The simultaneous uptake of both nitrogen sources, resulting in the most efficient energy source utilization, is in line with the experimental observation that plants exhibit optimal growth if nitrogen can be assimilated in both forms (${\text{NO}}_{3}^{-}$/${\text{NH}}_{4}^{+}$) [32, 33] and hints at underlying evolutionary optimization.

The model of Oliveira Dal’Molin *et al*. cannot account for this physiological feature because it lumps the light reactions, including FNR, into an artifcial reaction which, as stated by the authors, describes how “ATP and NADPH [are] generate[d] from light (overall reaction in chloroplast)”; to ensure ${\text{NO}}_{3}^{-}$ assimilation the FNR reaction is additionally implemented as a seperate reaction, thus rendering the light reactions and NO3- assimilation independent.

**Fig 3:**
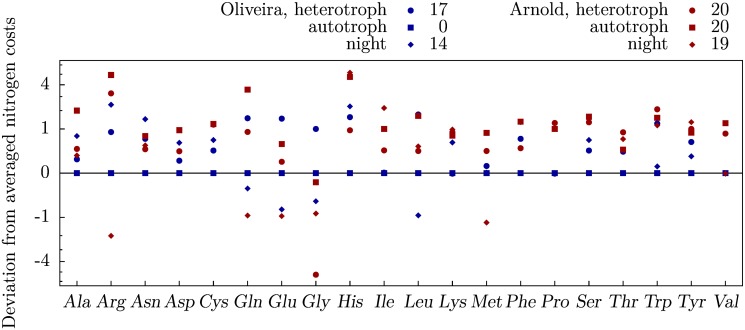
Deviation of costs for equal uptake of ${\text{NO}}_{3}^{-}$ and ${\text{NH}}_{4}^{+}$ from costs averaged over sole supply of the individual nitrogen sources (Δ*C*_N_). Shown are the deviations with respect to the models of de Oliveira Dal’Molin *et al*. [14] (blue), and Arnold and Nikoloski [15] (red). The values given in the legend denote the numbers of amino acids whose deviation differs from zero.

As mentioned above, the costs of amino acid synthesis increase with the ${\text{NO}}_{3}^{-}$ fraction of nitrogen uptake. Interestingly, this relation seems not to occur in a strictly proportional manner. For the majority of amino acids the deviation of the costs for equal uptake of ${\text{NO}}_{3}^{-}$ and ${\text{NH}}_{4}^{+}$ from the averaged costs for sole ${\text{NO}}_{3}^{-}$ uptake and sole ${\text{NH}}_{4}^{+}$ uptake,

$$\begin{array}{c} \Delta C_{N}\left(a_{i}\right)=\frac{1}{2}\left(C_{{\mathrm{NO}}_{3}}\left(a_{i}\right)+C_{{\mathrm{NH}}_{4}}\left(a_{i}\right)\right)-C_{50:50}\left(a_{i}\right), \end{array}$$ (3)

differs from zero (Fig. 3). The reason is likely to be found in the effect of the ratio of nitrogen source uptake on the operation of nitrate reductase (NR; EC 1.7.1.1) and NiR. Therefore, we expect that reactions linked via the other reactants of NR and NiR, namely ferredoxin and NAD(P)H, are also strongly affected. Indeed, we find a reciprocal relation of NiR to the presumably competing FNR such that less NADPH is produced if ${\text{NO}}_{3}^{-}$ is assimilated, which corroborates the proposed mechanism leading to mixed nitrogen source uptake under arbitrary uptake conditions. In addition, NADPH (or its unphosphorylated equivalent, NADH) is needed as a substrate of NR such that ${\text{NO}}_{3}^{-}$ assimilation induces further deficiency of NADPH. The complexity of the metabolic networks precludes identification of unique pathways. The exceptions to the nonzero deviation include the costs under autotrophic conditions of the model of de Oliveira Dal’Molin *et al*. which, as stated above, do not capture the competition of carbon fixation and ${\text{NO}}_{3}^{-}$ assimilation.

There is biological evidence for the various effects of nitrogen supply on metabolite levels, depending on the diurnal cycle and relations to the carbon supply. For instance, it was demonstrated for tobacco that the application of ${\text{NO}}_{3}^{-}$ fertilizers leads to increases in the rate of ${\text{NO}}_{3}^{-}$ uptake, levels of ${\text{NO}}_{3}^{-}$, rates of ${\text{NO}}_{3}^{-}$ assimilation, and the levels of ${\text{NH}}_{4}^{+}$, amino acids, and protein, ultimately resulting in an increase of growth rate [8, 34, 35]. Moreover, during the light period ${\text{NO}}_{3}^{-}$ decreases, and considerable amounts of ${\text{NH}}_{4}^{+}$ and of selected amino acids accumulate, especially glutamine, glycine and serine, with opposite behavior during the night. The effect of changes in the nitrogen supply is particularly pronounced for the levels of ${\text{NO}}_{3}^{-}$, ${\text{NH}}_{4}^{+}$ and selected amino acids such as glutamine and asparagine as well as on the ratios of glutamine and glutamate or asparagine and aspartate [36]. That said, mounting evidence has indicated that the effects of nitrogen supply and concentration of nitrogen-containing metabolites on growth are modulated via the coupling between carbon and nitrogen metabolisms, which calls for a large-scale analysis of metabolism. For instance, the increased supply of sugars can increase the rates of ${\text{NO}}_{3}^{-}$ and ${\text{NH}}_{4}^{+}$ uptake and assimilation, the synthesis of organic acid acceptors and the synthesis of amino acids. However, flux balance analysis, as a prominent method to analyze large-scale metabolic networks, cannot determine concentrations of metabolites. Therefore, in this framework it is challenging to make model-based predictions about the direct effects of nitrogen supply on the levels of amino acids and their implications on plant growth. However, amino acid synthesis costs, as quantified in this study, can add to the analysis of such observations, which bear strong relations to energy-related regulation.

## Conclusions

In this study, we have introduced an improved methodology to determine amino acid costs in terms of the energy equivalent ATP and have discussed the variation of individual costs due to the influence of external (and internal) perturbations. We have shown that the integration of environmental conditions has serious impact on the synthesis costs of amino acids in terms of energy: average weighted amino acid synthesis costs exhibited large dependence on the trophic level and on the nitrogen supply. Therefore, integration of environmental conditions is crucial in the analysis of the energy-balance related regulation in plants in order to arrive at meaningful interpretation.

**Fig 4:**
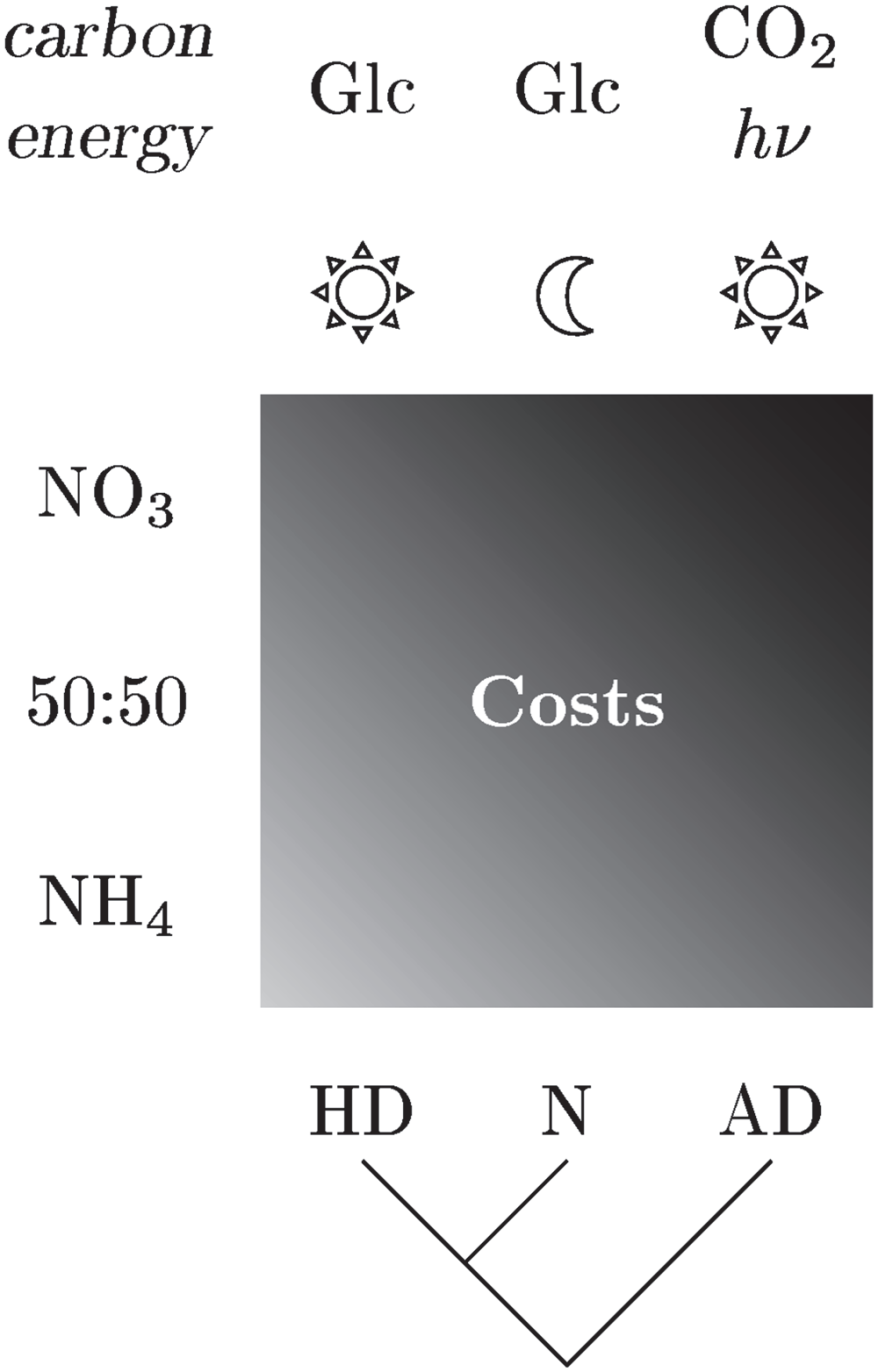
Schematic representation of the dependency of costs on the different investigated scenarios. Shown are the trends of amino acid synthesis costs for autotrophic and heterotrophic day (AD and HD) as well as night (N). The gradient represents the costs trend whereby lighter gray implies lower costs.

The majority of amino acid costs for night conditions are higher than those for heterotrophic day conditions. In addition, the costs for heterotrophic day conditions are distinctly exceeded by the costs for autotrophic conditions. While amino acid synthesis costs were systematically affected by the alternation of day and night, as well as of the trophic level (Fig. 4), positing hypotheses about resource reallocation in plants based on comparison of these costs warrants caution. The impact of metabolite synthesis costs in terms of energy depends on the availability of the respective resources (*e.g*., light intensity or on the amount of sugar storage): a limitation of resource availability imposes constraints on the energy budget. The effect of metabolite synthesis costs then depends on their ratio to the energy budget. Consequently, an environmental shift accompanied with a change in resource limitation may not only alter the metabolite synthesis costs but also the cellular energy budget and, hence, the effect of costs. The shift of the total energetic budget may then have important implications on resource reallocations.

The supply of nitrogen source was shown to significantly affect amino acid costs, exhibiting lowest costs for sole ${\text{NH}}_{4}^{+}$ and highest costs for sole ${\text{NO}}_{3}^{-}$ supply (Fig. 4, Table 2). The decrease in costs of amino acid synthesis for deviation from sole ${\text{NO}}_{3}^{-}$ supply towards an increase of ${\text{NH}}_{4}^{+}$ supply offers an explanation for the elevated amino acid and protein levels observed in plants grown under corresponding nitrogen supply conditions [11]. This is in line with former calculations showing increased production of enzymes for plants grown with ${\text{NH}}_{4}^{+}$ as sole nitrogen source compared to ${\text{NO}}_{3}^{-}$ [37].

We found enhanced efficiency of energy source utilization for mixed uptake of ${\text{NH}}_{4}^{+}$ and ${\text{NO}}_{3}^{-}$ which was observed in many plant species to result in optimal growth if both nitrogen sources were supplied [32, 33]. This may be the result of evolutionary optimization of metabolism balancing the assimilation of cheap but toxic ammonium and expensive but non-toxic nitrate. An accurate analysis of metabolic allocation of resources must carefully consider such effects, but cannot be conducted in the flux balance framework.

The utilization of resources often limits growth, even under optimal conditions [3]. Thus, resource allocation towards individual processes point at cellular strategies to ensure plant survival and reproduction. As protein synthesis, and therefore amino acid synthesis, consumes a major part of a plant’s available energetic resources [38], it is expected to play a considerable role in trade-offs arising from energetic limitation. Biological reasonable attribution of metabolite synthesis costs is a prerequisite to understand these trade-offs and their implications for plant growth. As it was also recently argued, metabolite synthesis costs (in terms of ATP) of ribulose 1,5-bisphosphate carboxylase oxygenase turnover represent a major investment in plants [15]. Therefore, the determination of condition-specific amino acid synthesis costs can serve as a starting point for metabolic engineering in plants towards improved growth and yield in agricultural applications. This could be achieved by manipulation of amino acid sequences of individual enzymes as well as rerouting whole pathways towards higher energetic efficiency of synthesis. However, understanding the energetic trade-offs may not only be exploited by using individually cheaper or more efficient pathways, but also by reorganization of metabolism. We believe that a deeper understanding of these trade-offs is an important step towards such holistic manipulations in plants.

## Material and Methods

### Flux Balance Analysis

Flux balance analysis (FBA) is a structural modeling framework developed to characterize the synthesizing capabilities of metabolic networks at steady state [17]. A metabolic network consists of *M* metabolites *X*_*i*_ (*i* = 1, …, *M*) and *N* reactions. The change of the concentration *x*_*i*_ of a metabolite *X*_*i*_ can be described as $\frac{\text{d}x_{i}}{\text{d}t}=\sum_{j}s_{ij}f_{j}-b_{i}$, where *s*_*ij*_ is the stoichiometric coefficient associated with the flux *f*_*j*_ through reaction *j* and *b*_*i*_ is the net transport flux of metabolite *X*_*i*_. The mass conservation relation under steady-state conditions, *i.e*., $\frac{\text{d}x_{i}}{\text{d}t}=0$, results in the following expression:

$$\begin{array}{c} S\cdot f-b=0, \end{array}$$ (4)

where **S** is the stoichiometric matrix (with *M* rows and *N* columns), **f** is the vector of metabolic fluxes of the *N* reactions and **b** is the vector representing consumption/production fluxes of the *M* metabolites. The consumption/production fluxes are set to zero for internal metabolites. In contrast, external metabolites constitute an interface to the environment and do not have to obey the steady-state condition. A metabolic flux crossing the system boundary is normally realized by a transporter reaction which converts an internal metabolite into an external metabolite. Constraints on the fluxes of the transporter reactions importing or exporting metabolites across the system boundary are utilized to establish environmental conditions, *e.g*., determining the availability of nutrients. As the system of equations described in (4) is usually under-determined (*N* > *M*), there exist multiple solutions corresponding to feasible flux distributions, each representing a particular metabolic state (with respect to fluxes) satisfying the stoichiometric constraints. The question usually addressed by FBA is that of determining which of the feasible metabolic states is manifested in the studied metabolic network.

FBA relies on the assumption that the metabolic system exhibits a state that is optimal with respect to some objective. Usually, the objective is expressed as a linear combination of fluxes in **f**, which leads to a linear programming problem:

$$\begin{array}{c} \text{min(max)}z=\sum_{i}c_{i}f_{i},\;\text{s.t.}\\ S\cdot f-b=0,\\ f_{min}\left(i\right)\leq f_{i}\leq f_{max}\left(i\right), \end{array}$$ (5)

with *z* representing the phenotypic property to be optimized, and **c** being a vector of coefficients quantifying the contribution of each flux to this objective. The bounds *f*_*min*_(*i*) and *f*_*max*_(*i*), represent constraints on the fluxes, *i.e*., the minimum and maximum values for the fluxes and, thus, determine reaction reversibility.

A common choice for the objective function is the maximization of biomass production, which allows a wide range of predictions consistent with experimental observations for simple model organisms [39–41] (an overview of objective functions is provided by Schuetz *et al*. [42]).

Calculations have been performed in the MATLAB environment using the CPLEX solver as part of the TOMLAB toolbox.

### Model modifications

To enable a reliable determination of amino acid costs, minor modifications for each model were made. To ensure a comparable framework, we first disabled all import and export as well as biomass reactions in the models and then enabled only the import and export reactions necessary for the examination of the individual scenarios (details are given in the next section). In the case of the models of Poolman *et al*. [13] and de Oliveira Dal’Molin *et al*. [14], additional modifications are required to enable the determination of the ATP production efficiency. De Oliveira Dal’Molin *et al*. provide an updated version of their model (http://web.aibn.uq.edu.au/cssb/resources/Genomes.html) which resolves the issue (S1 Table). For the model of Poolman *et al*., the authors do not provide an improved version. The authors stated that energy may be generated out of nothing due to unresolved imbalances with respect to protons and water [13]. This lead us to the assumption that there exists at least one futile cycle including the ATPase. To identify a futile cycle, we constrained energy source import by one and all other fluxes to be smaller than or equal to a much higher number, *e.g*., 1000 (reversible reactions were assigned the negative of that number as a lower boundary) and determined the maximum flux through the ATPase. We then consecutively knocked out each reaction of the network. Partaking of individual reactions in a futile cycle was indicated by a drastic reduction of ATP production upon their deletion (the algorithm is shown in S1 Fig). We manually gathered the participating reactions, defined a modification to cancel the futile cycle and integrated it in the succeeding iterations of the algorithm (modifications and futile cycles are shown in S1 and S2 Tables). Altogether, we identified all futile cycles affecting ATP production in three iterations, whereby analysis of single knockouts was sufficient.

For the compartmentalized models, we determined the minimum of amino acid costs across all compartments. In the model of Arnold and Nikoloski [15], the amino acid synthesis pathways are highly compartmentalized and, additionally, only a minimum number of transport reactions across the compartments exist. Therefore, for those amino acids with multiple synthesis pathway localizations, we determined compartment-specific costs. In contrast, for the model of de Oliveira Dal’Molin *et al*., all amino acid synthesis pathways are either completely located in the cytosol or transport reactions into the cytosol exist. Accordingly, implementation and utilization of export reactions for each amino acid from the cytosol is sufficient to determine the minimum amino acid costs in this model. However, the accuracy of costs, in this case, depends on the biological plausibility of the transport reactions in the original model.

In the model of Arnold and Nikoloski, there exist three maintenance reactions, one in each compartment (chloroplast, mitochondrion, and cytosol including the peroxisome). We determined that the maximum ATP production efficiency for the model can be achieved only if the sum of all maintenance reactions is taken into account. Accordingly, we checked whether similar effects can arise for the model of de Oliveira Dal’Molin *et al*. by incorporating maintenance reactions for each compartment. In doing so, we found the same ATP production efficiency with and without the additional reactions. Therefore, we used the original setup without additional maintenance reactions. The impact of an additional maintenance reaction for the model of Poolman *et al*. can be disregarded due to the lack of compartmentalization.

### Environmental and cellular set-up

**Fig 5:**
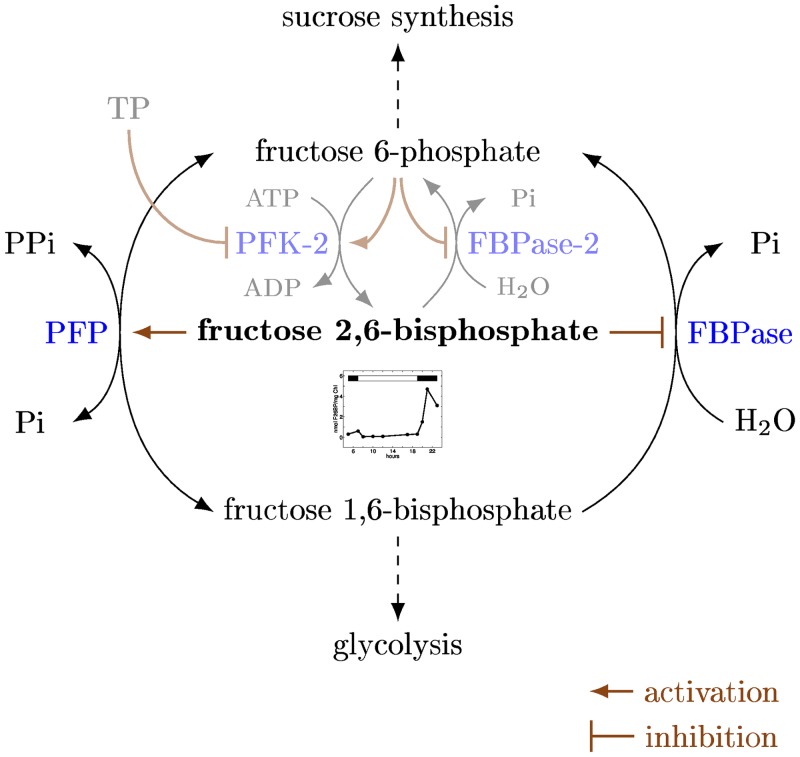
Schematic representation of the regulatory mechanism of fructose 2,6-bisphosphate (F26BP). The activity is regulated by the pyrophosphate-dependent phosphofructokinase (PFP) and the cytosolic fructose 1,6-bisphosphatase (FBPase) according to [43] and [44]. The regulators of F26BP itself are presented light-colored. The inlay graphic shows the regulatory effects of day-night alternation on the levels of F26BP (according to [46]).

A large percentage of plant metabolism is strongly affected by the change of day and night. In C_3_ plants, the predominant processes during the day include carbon fixation, photorespiration, and starch assimilation, opposed to starch degradation and cellular respiration (also referred to as dark respiration) at night. The majority of metabolism-related changes between day and night are attributed to the redox regulation of enzymes in the Calvin-Benson cycle, starch synthesis, ATP synthesis, and NADPH export from chloroplasts in response to light [23–26]. As structural metabolic modeling approaches cannot readily incorporate regulatory processes without making too many simplifying assumptions, we simulate the regulatory effect by scenario-dependent activation and deactivation of corresponding reactions. More precisely, we manipulate the ribulose 1,5-bisphosphate carboxylase oxygenase (RuBisCO; EC 4.1.1.39), the plastidic glyceraldehyde 3-phosphate dehydrogenases (GAPDH; EC 1.2.1.12 and 1.2.1.13), the plastidic fructose 1,6-bisphosphatase (FBPase; EC 3.1.3.11), sedoheptulose 1,7-bisphosphatase (SBPase; EC 3.1.3.37), the phosphoribulokinase (PRK; EC 2.7.1.19), the ADP-glucose pyrophosphorylase (AGPase; EC 2.7.7.27), the ATP synthase (EC 3.6.3.14), the NADP-linked malate dehydrogenase (NADP-MalDH; EC 1.1.1.82), and the phenylalanine ammonia-lyase (PAL; EC 4.3.1.24), the glucose 6-phosphate dehydrogenase (G6PDH; EC 1.1.1.49), and the ferredoxin-NADP reductase (FNR; EC 1.18.1.2). The details of the respective condition-specific modifications are given in S3 Table. In addition, we account for the regulatory effect of fructose 2,6-bisphosphate (F26BP) on the interconversion of fructose 6-phosphate (F6P) and fructose 1,6-bisphosphate (FBP) in the cytosol (the functioning of the mechanism is sketeched in Fig. 5 [43, 44]). From the modeling perspective, this results in a light dependent manipulation of the cytosolic FBPase and the pyrophosphate-dependent phosphofructokinase (PFP), respectively (S3 Table).

We investigate the impact of the trophic level, in particular autotrophic and heterotrophic growth conditions. The minimum nutrient requirements for autotrophic scenarios are usually the import of the low energy precursors H_2_O, CO_2_, Pi, ${\text{NO}}_{3}^{-}$ and/or ${\text{NH}}_{4}^{+}$, ${\text{SO}}_{4}^{2-}$ and/or H_2_S in addition to light as energy source. In contrast, in heterotrophic scenarios the carbon source, here Glc, serves simultaneously as energy source. In that case, to preclude the carbon fixation and utilization of light energy, we disabled the photosystem reactions and deactivated the CO_2_ import (S3 Table). For the analysis of amino acid synthesis in this study, the number of precursors can be further reduced. In addition, Pi is not required and the model of de Oliveira Dal’Molin *et al*. [14] cannot handle ${\text{SO}}_{4}^{2-}$. The respective importers were therefore disabled. An additional importer had to be implemented in the model of Poolman *et al*. [13] to enable utilization of H_2_S as sulfur source across all models (S3 Table).

### Calculation of amino acid synthesis cost

The calculation of amino acid synthesis costs is based on flux balance analysis. The cost calculation requires the utilized metabolic reconstruction to have importers of nutrients and exporters of amino acids. Additionally, our approach requires a maintenance reaction (*i.e*., a generic ATP hydrolysis reaction, ATP → ADP + Pi) which accounts for generic energy consuming processes. For the models in which any of these reactions is not present, it was additionally included. The model of Arnold and Nikoloski [15] comprises a maintenance reaction for every compartment. Therefore, the flux through the maintenance reaction is in fact the sum of fluxes carried by all three compartment-specific maintenance reactions. Constraints according to the examined model, the environmental conditions and the trophic level are referred to as initial constraints (shown in S3 Table).

**Fig 6:**
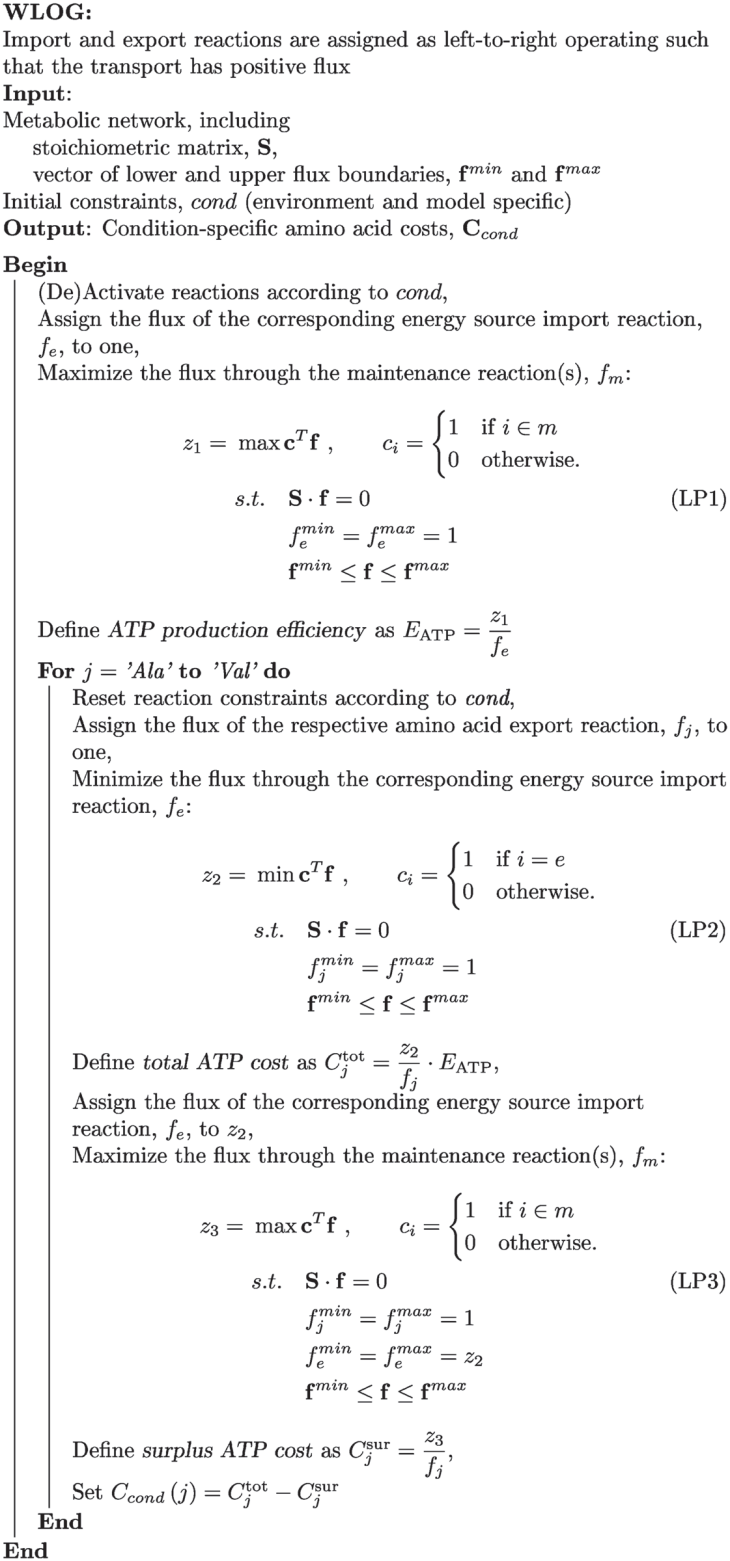
Algorithm for the computation of condition-specific amino acid costs.

The amino acid cost calculation proceeds in three steps. We assume that nutrient import, amino acid export and the maintenance reaction(s) are such that they carry nonnegative flux. First, we determine the ATP production efficiency, as described in the algorithm shown in Fig. 6 (LP1): the initial constraints are imposed, the energy source import, *f*_*e*_, is constrained to 1 and the flux through the maintenance reaction(s) is maximized. The ATP production efficiency gives the maximum number of ATP molecules which can be produced per imported unit of energy source. It has the dimension [ATP molecules/energy source unit]. The ATP production efficiency is a property of the network and the initial constraints.

The amino acid cost calculation is then executed according to the two following steps, which are repeated for every amino acid.

After the initial constraints have been restored, the total ATP cost, $C_{j}^{tot}$, is determined, as described in the algorithm presented in Fig. 6 (LP2): the export of the considered amino acid is set to 1 and the import of the energy source is minimized. Multiplying the minimal energy source import per amino acid export with the ATP production efficiency of the energy source yields the total ATP cost, $C_{j}^{tot}$, of amino acid synthesis with dimension [ATP molecules/amino acid molecule]. Therefore, the total ATP cost quantifies the maximum amount of ATP that could be produced from the energy source instead of synthesizing the amino acid using the pathway with minimum energy source requirement.

In the third step, we determine the surplus ATP cost, $C_{j}^{sur}$, as described in the algorithm shown in Fig. 6 (LP3): we additionally fix the (minimum) energy source import and maximize the flux through the maintenance reaction(s). The ratio of the flux through the maintenance reaction(s) and the amino acid import yields the surplus ATP cost, $C_{j}^{sur}$, with dimension [ATP molecules/amino acid molecule].

The amino acid synthesis cost then is given as the difference of the total and the surplus ATP cost.

It should be noted that the amino acid costs depend on the energy source uptake or on the amino acid export flux, if nonzero boundaries of other reactions limit the outcome of the optimization in any of the three steps. This is usually not the case in the original setup of metabolic reconstructions (like in this study), but can occur when additional boundary conditions are incorporated, *e.g*., by utilizing high-throughput data [45].

### Models

We provide the models with the denoted modifications in the SBML format in the Supplementary S2 dataset. The majority of modifications have been implemented as changes of the reaction boundaries.

## Supporting Information

S1 Fig Algorithm for the detection of futile cycles in the model of Poolman *et al*. [13]. (TIF)

S1 Table Model adjustments. Modified model constraints compared to the published versions of the models of Poolman *et al*. [13] and de Oliveira Dal’Molin *et al*. [14] which are valid for all scenarios. Lower and upper boundaries are denoted by *lb* and *ub*, respectively.
(PDF)

S2 Table Futile cycles in the model of Poolman *et al*.. The reactions marked with ‘>’ were detected by the Algorithm shown in S1 Fig, and the reactions marked with ‘•’ are revised reactions (the respective adaption is given in S1 Table). ‘∘’ denotes reactions which are presented in a reversed manner compared to their implemented annotation (reads now right-to-left). The metabolite annotation is in accordance to that of Poolman *et al*..
(PDF)

S3 Table Boundary constraints for autotrophic and heterotrophic day (Aut and Het), and night (Nig) conditions. As the model of Poolman *et al*. [13] is only capable to simulate the heterotrophic day and the night scenario, the entries for the autotrophic day scenario pertain exclusively to the models of de Oliveira Dal’Molin *et al*. [14], and Arnold and Nikoloski [15]. Due to the incapability of the model of de Oliveira Dal’Molin *et al*. [14] to utilize ${\text{SO}}_{4}^{2-}$, a H_2_S importer is implemented for the model of Poolman *et al*. (^a^), and the ${\text{SO}}_{4}^{2-}$ importer is disabled (^b^). The unnecessary import of Pi for amino acid synthesis is deactivated (^c^).
(PDF)

S1 Dataset Amino acid synthesis costs for all examined models and conditions; amino acid frequencies based on the dataset of Mooney *et al*. [20]. (XLS)

S2 Dataset Metabolic network models in SBML format. (ZIP)
