# Supplementary figures and images for: Effects of Varying Nitrogen Sources on Amino Acid Synthesis Costs in Arabidopsis thaliana under Different Light and Carbon-Source Conditions
Source: PLoS One. 2015 Feb 23;10(2):e0116536. doi: 10.1371/journal.pone.0116536 (PMC4338252; doi:10.1371/journal.pone.0116536)

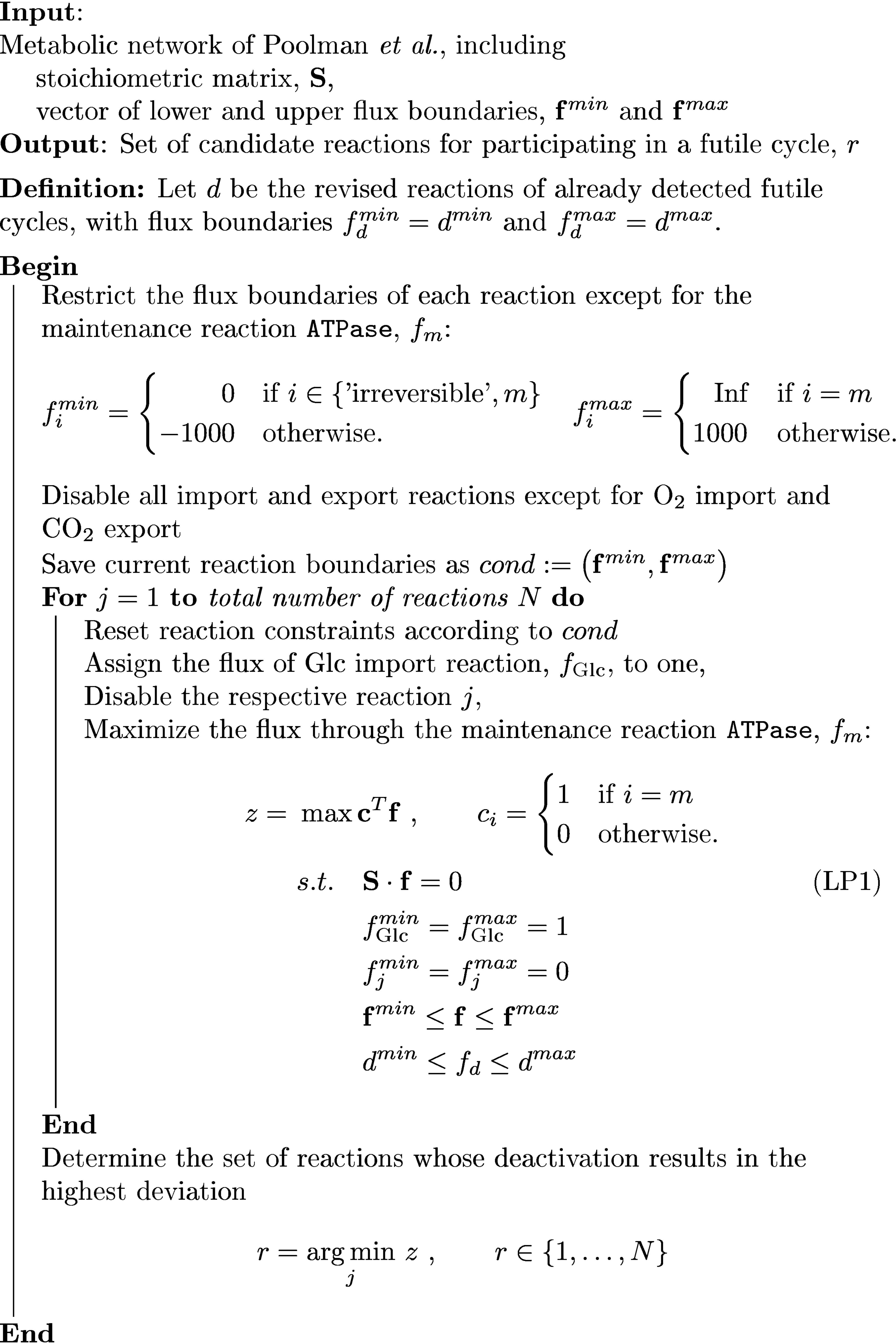

Supplement: S1 Fig — (TIF) [file pone.0116536.s001.tif]
