# Supplementary material for: Effects of Varying Nitrogen Sources on Amino Acid Synthesis Costs in Arabidopsis thaliana under Different Light and Carbon-Source Conditions
Source: PLoS One. 2015 Feb 23;10(2):e0116536. doi: 10.1371/journal.pone.0116536 (PMC4338252; doi:10.1371/journal.pone.0116536)
Supplement: S1 Table — Modified model constraints compared to the published versions of the models of Poolman et al. [13] and de Oliveira Dal’Molin et al. [14] which are valid for all scenarios. Lower and upper boundaries are denoted by lb and ub, respectively. (PDF) [file pone.0116536.s002.pdf]

Table S1. Model adjustments.

Model	Reaction ID	*lb*	*ub*
Poolman	`reac_1321`	0	Inf
	`reac_1331`	0	Inf
	`MalDH`	0	v(`AlphaKGDH`)
de Oliveira	`TCM22`	0	0
	`TCP27`	0	0
	`TCX14`	-Inf	Inf
	`R00086_c`	0	Inf
	`R00093_p`	0	0
	`R00114_c`	0	0
	`R00149_p`	0	0
	`R00243_c`	0	Inf
	`R00243_m`	0	Inf
	`R00253_m`	0	Inf
	`R00253_p`	0	Inf
	`R00343_m`	0	0
	`R00472_x`	0	Inf
	`R00945_c`	0	0
	`R01221_m`	0	0
	`R01398_c`	0	0
	`R01752_c`	0	0
	`R05875_p`	0	0

Modified model constraints compared to the published versions of the models of Poolman *et al.* [7] and de Oliveira Dal'Molin *et al.* [8] which are valid for all scenarios. Lower and upper boundaries are denoted by *lb* and *ub*, respectively.
