# Supplementary material for: Effects of Varying Nitrogen Sources on Amino Acid Synthesis Costs in Arabidopsis thaliana under Different Light and Carbon-Source Conditions
Source: PLoS One. 2015 Feb 23;10(2):e0116536. doi: 10.1371/journal.pone.0116536 (PMC4338252; doi:10.1371/journal.pone.0116536)
Supplement: S2 Table — The reactions marked with ‘>’ were detected by the Algorithm shown in S1 Fig, and the reactions marked with ‘•’ are revised reactions (the respective adaption is given in S1 Table). ‘∘’ denotes reactions which are presented in a reversed manner compared to their implemented annotation (reads now right-to-left). The metabolite annotation is in accordance to that of Poolman et al.. (PDF) [file pone.0116536.s003.pdf]

**Table S2.** Futile cycles in the model of Poolman *et al.*.

| Reaction ID | Reaction formula | | | Note |
|---|---|---|---|---|
| reac_374 | 2 GSH + NADP | $\rightarrow$ | NADPH + GSSG | |
| reac_33 | AMP + $SO_3$ + GSSG | $\rightleftharpoons$ | 2 GSH + APS | ○ |
| reac_1331 | PPi + APS | $\rightleftharpoons$ | $SO_4$ + ATP | ● ○ |
| reac_1317 | 2 Cyt$c_{red}$ + $SO_4$ | $\rightarrow$ | 2 Cyt$c_{ox}$ + $SO_3$ | |
| > reac_267 | 2 Cyt$c_{ox}$ + Lac | $\rightleftharpoons$ | 2 Cyt$c_{red}$ + Pyr | |
| > reac_302 | Pyr + NADH | $\rightleftharpoons$ | NAD + Lac | ○ |
| 1. | AMP + PPi + NADH + NADP | $\rightarrow$ | ATP + NAD + NADPH | |
| reac_1260 | NADH + *Glu* | $\rightleftharpoons$ | NAD + P5C | ○ |
| > reac_1321 | P5C | $\rightleftharpoons$ | *Glu*-SeA | ● ○ |
| reac_379 | *Glu*-SeA + NADP + Pi | $\rightleftharpoons$ | NADPH + *Glu*P | |
| reac_378 | *Glu*P + ADP | $\rightleftharpoons$ | *Glu* + ATP | ○ |
| 2. | ADP + Pi + NADH + NADP | $\rightleftharpoons$ | ATP + NAD + NADPH | |
| FumEquiv | FUM | $\rightleftharpoons$ | Fum | ○ |
| Fumarase | Fum + $H_2O$ | $\rightleftharpoons$ | Mal | |
| > MalDH | Mal + $NAD_m$ | $\rightleftharpoons$ | OxalAc + $NADH_m$ + $H_m$ | ● |
| OAAEquiv | OxalAc | $\rightleftharpoons$ | OAA | |
| reac_197 | OAA + *Glu* | $\rightleftharpoons$ | KG + *Asp* | ○ |
| reac_363 | $NH_3$ + KG + NADH | $\rightleftharpoons$ | NAD + *Glu* | ○ |
| reac_158 | GTP + IMP + *Asp* | $\rightarrow$ | GDP + DC-AMP + Pi | |
| reac_180 | DC-AMP | $\rightarrow$ | FUM + AMP | |
| reac_178 | AMP | $\rightarrow$ | $NH_3$ + IMP | |
| reac_347 | GDP + ATP | $\rightleftharpoons$ | GTP + ADP | |
| Complex_I | $NADH_m$ + Q + 5 $H_m$ | $\rightarrow$ | $NAD_m$ + $QH_2$ + 4 H | |
| Complex_III | $QH_2$ + 2 Cyt$c_{ox}$ + 2 $H_m$ | $\rightarrow$ | Q + 2 Cyt$c_{red}$ + 4 H | |
| > Complex_IV | 4 Cyt$c_{red}$ + 8 $H_m$ + $O_2$ | $\rightarrow$ | 4 Cyt$c_{ox}$ + 2 $H_2O$ + 4 H | |
| > Complex_V | 4 H + ADP + Pi | $\rightarrow$ | 4 $H_m$ + ATP | |
| 3. | $\frac{3}{2}$ ADP + $\frac{3}{2}$ Pi + $\frac{1}{2}$ $O_2$ + NADH | $\rightarrow$ | $\frac{3}{2}$ ATP + NAD | |

The reactions marked with '>' were detected by the Algorithm shown in Figure 5, and the reactions marked with '●' are revised reactions (the respective adaption is given in Table S1). '○' denotes reactions which are presented in a reversed manner compared to their implemented annotation (reads now right-to-left). The metabolite annotation is in accordance to that of Poolman *et al.*.
