# Supplementary material for: Effects of Varying Nitrogen Sources on Amino Acid Synthesis Costs in Arabidopsis thaliana under Different Light and Carbon-Source Conditions
Source: PLoS One. 2015 Feb 23;10(2):e0116536. doi: 10.1371/journal.pone.0116536 (PMC4338252; doi:10.1371/journal.pone.0116536)
Supplement: S3 Table — As the model of Poolman et al. [13] is only capable to simulate the heterotrophic day and the night scenario, the entries for the autotrophic day scenario pertain exclusively to the models of de Oliveira Dal’Molin et al. [14], and Arnold and Nikoloski [15]. Due to the incapability of the model of de Oliveira Dal’Molin et al. [14] to utilize SO42−, a H2S importer is implemented for the model of Poolman et al. (a), and the SO42− importer is disabled (b). The unnecessary import of Pi for amino acid synthesis is deactivated (c). (PDF) [file pone.0116536.s004.pdf]

Reaction	Arnold				de Oliveira				Poolman			Refs
	ID	Aut	Het	Nig	ID	Aut	Het	Nig	ID	Het	Nig	
Glucose exchange	Ex_Glc	–	$\leftarrow$	$\leftarrow$	Ex13	–	$\leftarrow$	$\leftarrow$	GLC_tx	$\rightarrow$	$\rightarrow$	
Photon exchange	Im_hnu	$\rightarrow$	$\rightarrow$	–	Ex16	$\rightarrow$	$\rightarrow$	–	–			
CO_2 exchange	Im_CO2	$\rightleftharpoons$	$\leftarrow$	$\leftarrow$	Ex1	$\rightleftharpoons$	$\leftarrow$	$\leftarrow$	CO2_tx	$\rightarrow$	$\rightarrow$	
H_2O exchange	Im_H2O	$\rightleftharpoons$	$\rightleftharpoons$	$\rightleftharpoons$	Ex2	$\rightleftharpoons$	$\rightleftharpoons$	$\rightleftharpoons$	–			
O_2 exchange	Ex_O2	$\rightleftharpoons$	$\rightleftharpoons$	$\rightleftharpoons$	Ex3	$\rightleftharpoons$	$\rightleftharpoons$	$\rightleftharpoons$	O2_tx	$\rightleftharpoons$	$\rightleftharpoons$	
NO_3^- exchange	Im_NO3	$\rightarrow$	$\rightarrow$	$\rightarrow$	Ex4	$\rightarrow$	$\rightarrow$	$\rightarrow$	NO3_tx	$\rightarrow$	$\rightarrow$	
NH_4^+ exchange	Im_NH4	$\rightarrow$	$\rightarrow$	$\rightarrow$	Ex5	$\rightarrow$	$\rightarrow$	$\rightarrow$	NH3_tx	$\rightarrow$	$\rightarrow$	
H_2S exchange	Im_H2S	$\rightarrow$	$\rightarrow$	$\rightarrow$	Ex11	$\rightarrow$	$\rightarrow$	$\rightarrow$	Im_H2S [a]	$\rightarrow$	$\rightarrow$	
SO_4^{2-} exchange [b]	Im_SO4	–	–	–	Ex12	–	–	–	SO4_tx	–	–	
Pi exchange [c]	Im_Pi	–	–	–	Ex18	–	–	–	Pi_tx	–	–	
Photosystems	PSII_h	$\rightarrow$	–	–	REner01_p	$\rightarrow$	–	–				
	PSI_h	$\rightarrow$	–	–								
FNR	FdNADPR_h	$\rightarrow$	$\rightleftharpoons$	$\rightleftharpoons$	R01195_p	$\rightleftharpoons$	$\rightleftharpoons$	$\rightleftharpoons$	–			[25, 47]
ATP synthase	ATPase_h	$\rightleftharpoons$	$\rightleftharpoons$	–	REner01_p	$\rightarrow$	–	–	–			[26]
RuBisCO	RBC_h	$\rightarrow$	$\rightarrow$	–	R00024_p	$\rightarrow$	$\rightarrow$	–	reac_621	$\rightarrow$	–	[48]
	RBO_h	$\rightarrow$	$\rightarrow$	–	R03140_p	$\rightarrow$	$\rightarrow$	–	reac_1136	$\rightarrow$	$\rightarrow$	
GAPDH (plast)	GAPDH1_h	$\rightarrow$	$\rightarrow$	–	R01061_p	$\rightleftharpoons$	$\rightleftharpoons$	–	reac_17	$\rightarrow$	–	[26, 48]
	GAPDH2_h	$\rightleftharpoons$	$\rightleftharpoons$	–	R01063_p	$\rightleftharpoons$	$\rightleftharpoons$	–				
FBPase	FBPase_h	$\rightarrow$	$\rightarrow$	–	R00762_p	$\rightarrow$	$\rightarrow$	–	reac_318	$\rightarrow$	–	[26, 48]
					R04780_p	$\rightarrow$	$\rightarrow$	–				
	FBPase_c	$\rightarrow$	$\rightarrow$	–	R00762_c	$\rightarrow$	$\rightarrow$	–				[43, 44]
					R04780_c	$\rightarrow$	$\rightarrow$	–				
SBPase	SBPase_h	$\rightarrow$	$\rightarrow$	–	R01845_p	$\rightarrow$	$\rightarrow$	–	reac_1305	$\rightarrow$	–	[26, 48]
PRK	Ru5PK_h	$\rightarrow$	$\rightarrow$	–	R01523_p	$\rightarrow$	$\rightarrow$	–	reac_562	–	$\rightarrow$	[26, 48]
AGPase	AGPase_h	$\rightarrow$	$\rightarrow$	–	R00948_p	$\rightleftharpoons$	$\rightleftharpoons$	–	reac_354	$\rightarrow$	–	[24]
NADP-MalDH (plast)	MalDH3_h	$\rightarrow$	$\rightarrow$	–	R00343_p	$\rightleftharpoons$	$\rightleftharpoons$	–	–			[26, 48]
G6PDH	G6PDH_h	–	–	$\rightarrow$	R02736_p	–	–	$\rightarrow$	reac_353	–	$\rightarrow$	[26, 48]
PFP	PPIF6PK_c	–	–	$\rightleftharpoons$	–				reac_77	–	$\rightarrow$	[43, 44]
PAL (cyt)	–				R00697_c	$\rightarrow$	$\rightarrow$	–	reac_555	$\rightarrow$	–	[48]

As the model of Poolman *et al.* [13] is only capable to simulate the heterotrophic day and the night scenario, the entries for the autotrophic day scenario pertain exclusively to the models of de Oliveira Dal'Molin *et al.* [14], and Arnold and Nikoloski [15]. Due to the incapability of the model of de Oliveira Dal'Molin *et al.* [14] to utilize SO_4^{2-}, a H_2S importer is implemented for the model of Poolman *et al.* ([a]), and the SO_4^{2-} importer is disabled ([b]). The unnecessary import of Pi for amino acid synthesis is deactivated ([c]).
